# Supplementary material for: Measuring competing outcomes of a single-molecule reaction reveals classical Arrhenius chemical kinetics
Source: Nat Commun. 2024 Nov 28;15:10322. doi: 10.1038/s41467-024-54677-1 (PMC11604936; doi:10.1038/s41467-024-54677-1)
Supplement: Supplementary file 1 — Supplementary Information [file 41467_2024_54677_MOESM1_ESM.pdf]

Supplementary Information for

Measuring competing outcomes of a single-molecule reaction reveals classical Arrhenius chemical kinetics

Pieter J. Keenan[1, 2, 3, †], Rebecca M. Purkiss[1, †], Tillmann Klamroth[4], Peter A. Sloan[1,2], Kristina R. Rusimova[1,2,3, *]

[1]Department of Physics, University of Bath, Bath, BA2 7AY, UK.

[2]Centre for Nanoscience and Nanotechnology, University of Bath, Bath, BA2 7AY, UK.

[3]Centre for Photonics and Photonic Materials, University of Bath, Bath, BA2 7AY, UK.

[4]Universit ät Potsdam, Institut für Chemie, Theoretische Chemie, D-14476, Potsdam, Germany.

†These authors contributed equally to this work.

*Corresponding author. Email: k.r.rusimova@bath.ac.uk

Contents:

Supplementary Note 1. Details of single-molecule manipulation measurement
Supplementary Note 2. Alternative analysis for manipulation rates and branching ratios
Supplementary Note 3. Discussion of manipulation branching ratio data at 2.1 V
Supplementary Note 4. Manipulation probability tree
Supplementary Note 5. Density functional theory calculations: optimised geometries
Supplementary Note 6. Density functional theory calculations: transition barriers

Supplementary Note 1. Details of single-molecule manipulation measurement

Toluene is di-σ bonded to the Si(111)-7×7 surface, forming one covalent bond to a silicon adatom and another to a neighbouring silicon restatom Suppl. Ref. [1]. Previously we reported on the local manipulation of the toluene/Si(111)-7×7 system [2]. For electron injection into molecules adsorbed to faulted middle adatoms we found two populations depending on their precise chemisorption geometry (choice of two crystollographically equivalent rest-atoms). This resulted in two rates of desorption, dependent upon whether the tip was atop the centre of the molecule, or more atop the position of the bonding adatom. The former gave a higher rate of manipulation than the latter. Instead, here we perform injections into faulted corner adatoms where there is only one possible molecular bonding configuration, resulting in a single rate of desorption.

Suppl. Fig. 1 shows the Δz_M heights (see Fig. 1) for injection data collected at -1.2 V and 20 pA, which informed our choice of 30 pm as an appropriate value at which to trigger the stopping of the injections. The variation in the data points of Suppl. Fig. 1 may be attributed to different tip states or to slight variation in the injection site position still occasionally present during injections.

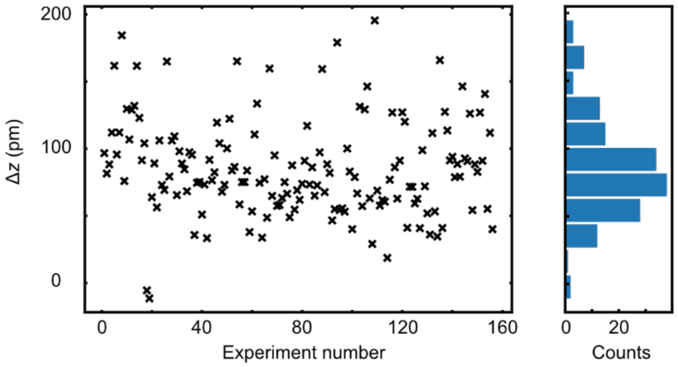

Supplementary Figure 1 Distribution of measured changes in tip height resulting from molecular manipulation. Measured Δz_M for -1.2 V and 20 pA injections and corresponding histogram of heights.

Supplementary Note 2. Alternative analysis for manipulation rates and branching ratios

An alternative way of calculating the branching ratio is to fit to the individual rates for desorption and switching and then take the ratio of these rates, rather than the random walk method shown in the main paper. To do this, we first fit to the total probability of manipulation $P(t) = \frac{N_{\mathrm{manip}}}{N_0} = 1 - \exp(-kt)$. Then for each outcome, we fit to $P_x = \frac{N_x}{N_0} = \frac{k_x}{k}\left(1 - e^{-kt}\right)$, with *x* either d for desorption or s for switching, N_0 the number of attempted molecular manipulation events, and N_{manip} corresponding to the total number of manipulated molecules (both desorbed and switched). Suppl. Figure 2 shows the fits of the data this way. The branching ratio is again given by $B = {k_{\mathrm{d}}}/{k_{\mathrm{s}}}$. This fitting procedure gives similar results for both the manipulation rates and reaction branching ratios as the ones in the paper, but requires an additional fitting parameter. Thus, the method in the main text is preferred.

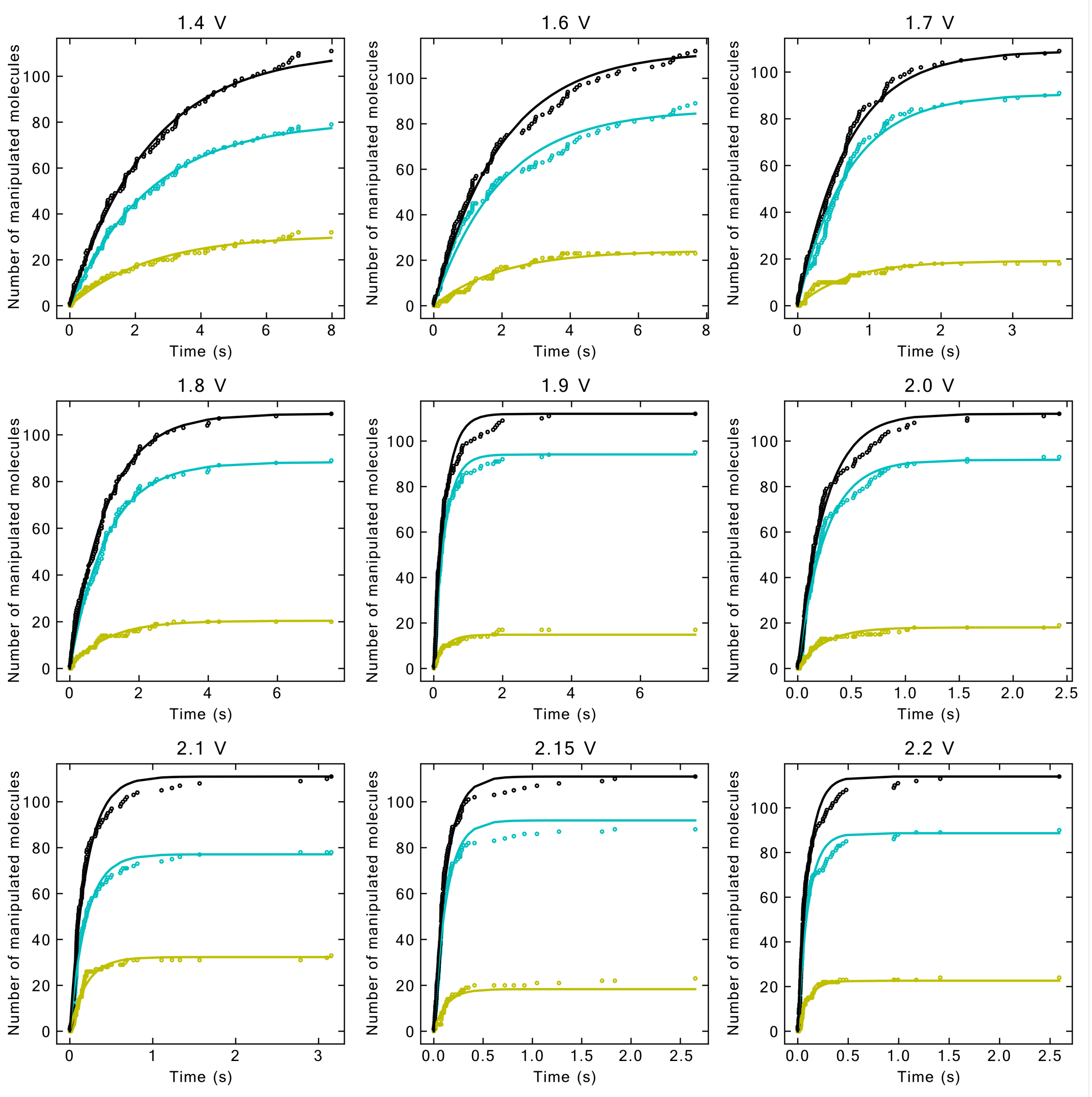

Supplementary Figure 2 Alternative method for extracting manipulation probabilities. Solid lines correspond to the fits. Black: both manipulation outcomes combined; cyan: desorption; yellow: diffusion.

Supplementary Note 3. Discussion of manipulation branching ratio data at 2.1 V

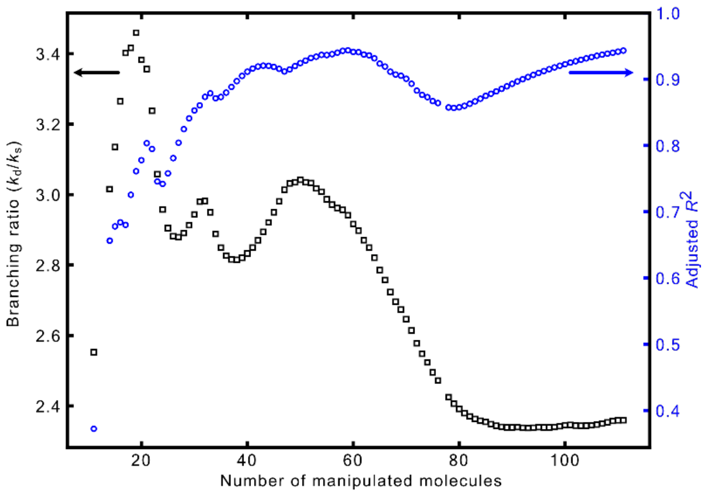

Supplementary Figure 3 Manipulation branching ratio as a function of number of manipulated molecules. Calculated branching ratio k_d/k_s and adjusted R^2 value of the fit as a function of number of manipulated molecules for injections at 2.1 V and 750 pA.

Suppl. Figure 3 shows the calculated branching ratio k_d/k_s and adjusted R^2 parameter of the linear fit as a function of number of manipulated molecules. Depending on the number of molecules that are taken into account for the fit, there appear to be two distinct populations with high R^2 values but resulting in two different values for the branching ratio. This could be an artifact of the presence of two dominating tip states when this dataset was present. Therefore, the error bar presented in the main paper is an underestimate and the data point in Figure 5a may not be a true measurement of the branching ratio at this injection bias voltage. In addition, measurements above the nonlocal manipulation threshold at ≈ 2.0 V are much more challenging due to the short measured time to manipulation.

Supplementary Note 4. Manipulation probability tree

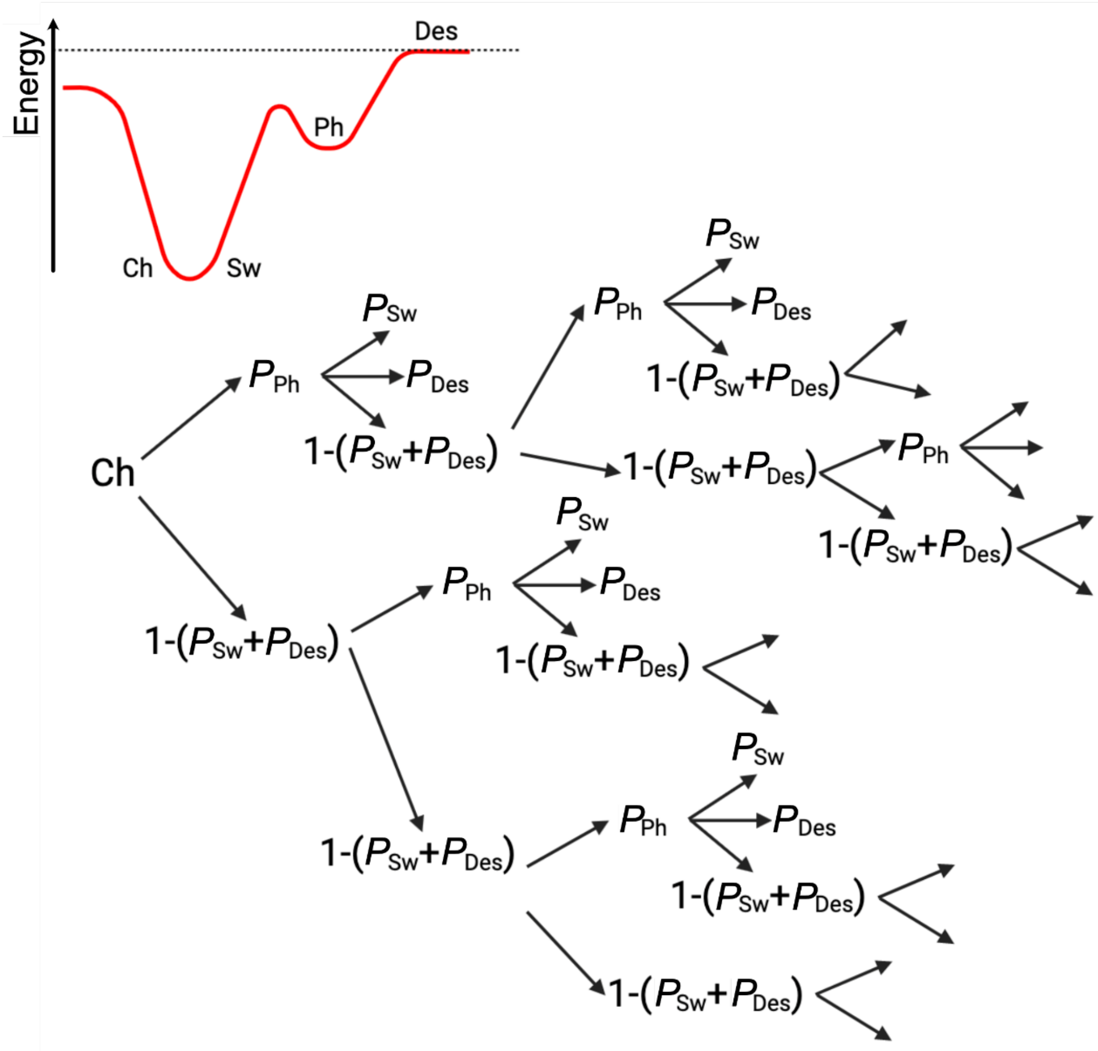

Supplementary Figure 4 Reaction outcome probability tree. Reaction outcome probability tree for a single chemisorbed molecule. Inset: Ground state molecular potential indicating the labelling scheme.

Suppl. Fig. 4 shows the manipulation probability tree for outcomes that include the reattachment of the molecule in its initial chemisorbed configuration, following excitation. By using the probability tree, we identify that the fraction of molecules reaching each observable outcome is given by the following set of expressions, where *N* denotes the number of molecules in each final state, N_0 is the total initial number of molecules, and *P* is the probability of each outcome, with subscripts Ph, Sw, Des, Ch corresponding to physisorbed, switched, desorbed, and chemisorbed.

$$\text{Switched} \Rightarrow \frac{N_{\mathrm{Sw}}}{N_0} = P_{\mathrm{Ph}}P_{\mathrm{Sw}} + P_{\mathrm{Ph}}[1-(P_{\mathrm{Sw}}+P_{\mathrm{Des}})]P_{\mathrm{Ph}}P_{\mathrm{Sw}} +$$
$$+P_{\mathrm{Ph}}[1-(P_{\mathrm{Sw}}+P_{\mathrm{Des}})]P_{\mathrm{Ph}}[1-(P_{\mathrm{Sw}}+P_{\mathrm{Des}})]P_{\mathrm{Ph}}P_{\mathrm{Sw}} + \cdots$$
$$+(1-P_{\mathrm{Ph}})P_{\mathrm{Ph}}P_{\mathrm{Sw}} + ------- '' ------ \qquad \text{(S1)}$$
$$+(1-P_{\mathrm{Ph}})^2P_{\mathrm{Ph}}P_{\mathrm{Sw}} + ------ '' ------$$
$$+(1-P_{\mathrm{Ph}})^3P_{\mathrm{Ph}}P_{\mathrm{Sw}} + ------ '' ------$$

$$\frac{N_{\mathrm{Sw}}}{N_0} = \sum_{n=0}^{\infty}(1-P_{\mathrm{Ph}})^n P_{\mathrm{Ph}}P_{\mathrm{Sw}}[1-(P_{\mathrm{Sw}}+P_{\mathrm{Des}})]^n P_{\mathrm{Ph}}^n \qquad \text{(S2)}$$

By the same analysis:

$$\text{Desorbed} \Rightarrow \frac{N_{\text{Des}}}{N_0} = \sum_{n=0}^{\infty}(1 - P_{\text{Ph}})^n P_{\text{Ph}} P_{\text{Des}} [1 - (P_{\text{Sw}} + P_{\text{Des}})]^n P_{\text{Ph}}^n \quad \text{(S3)}$$

Using the expression for an infinite geometric series, $\sum_{k=0}^{\infty} ar^k = \frac{1}{1-r}; |r| < 1$, and identifying $a = P_{\text{Ph}} P_{\text{Sw/Des}}$ and $r = (1 - P_{\text{Ph}})^n [1 - (P_{\text{Sw}} + P_{\text{Des}})]^n P_{\text{Ph}}^n$ the above expressions simplify to:

$$\frac{N_{\text{Sw}}}{N_0} = \frac{P_{\text{Ph}} P_{\text{Sw}}}{1-(1-P_{\text{Ph}})^n [1-(P_{\text{Sw}}+P_{\text{Des}})]^n P_{\text{Ph}}^n} \quad \text{(S4)}$$

and

$$\frac{N_{\text{Des}}}{N_0} = \frac{P_{\text{Ph}} P_{\text{Des}}}{1-(1-P_{\text{Ph}})^n [1-(P_{\text{Sw}}+P_{\text{Des}})]^n P_{\text{Ph}}^n} \quad \text{(S5)}$$

From the ratio of these we can recover $\frac{N_{\text{Des}}}{N_{\text{Sw}}} = \frac{P_{\text{Des}}}{P_{\text{Sw}}}$, which is identical to the expression without considering molecular reattachment pathway.

Supplementary Note 5. Density functional theory calculations: optimised geometries

The system of toluene on the Si(111)−7×7 surface was investigated with the quantum chemical cluster models developed in Suppl. Refs. [3,4]. Model A of the Si(111)−7×7 surface comprises 17 silicon atoms and 26 saturating hydrogen atoms, while model B is slightly larger (21 silicon, 34 hydrogen atoms) as it contains all silicon atoms up to two bonds distance from the adatom-restatom pair that are responsible for the bonding to toluene. The nomenclature used to label atoms and methyl group substitution positions within the cluster is defined in Suppl. Fig. 5. In previous work, we showed that the clusters used in these calculations (A and B) are sufficient to describe the behaviours of the system and that they perform well in comparison to a much larger and more computationally expensive model (67 silicon, 54 hydrogen atoms) [4].

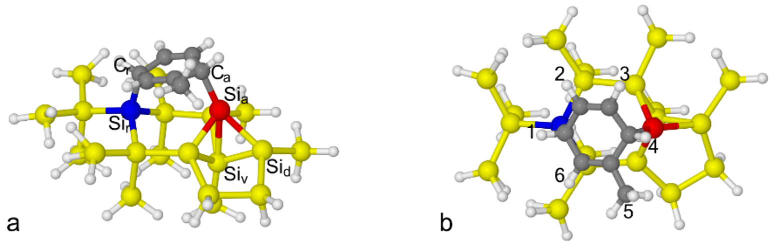

Supplementary Figure 5 Cluster model and nomenclature. For the B-PhCH3 cluster, a) presents labels used for selected atoms and b) shows the numbering scheme for possible substitution positions (R-positions) of the methyl group. The silicon adatom (Si_a) is coloured red, the restatom (Si_r) blue, all other silicon atoms are yellow while carbon atoms are grey and hydrogen atoms white. The carbon atoms bonded to the adatom and the restatom are denoted as C_a and C_r. The out-of-plane movement of the adatom in the anionic states is characterised by the distances to the atoms labelled Si_v and Si_d.

Quantum chemical cluster model geometry optimization calculations were performed using the B3LYP hybrid functional, employing the 6-31G* basis set and the Grimme D3 dispersion correction applied as previously done in Suppl. Ref. [4]. The neutral ground state (neutral), the first excited state of the negatively charged system (anion*) and its ground state (anion) were optimised for the A-$PhCH_3$ and B-$PhCH_3$ cluster models, for both the R3 and R5 functional group positions (detailed in Suppl. Fig. 5b). The optimised geometries for A-$PhCH_3$-R3 are shown in Suppl. Fig. 6 and the inter-atomic distances for selected atoms are presented in Suppl. Table 1. It was found that the geometry of the anion* state is between those of the neutral and anion systems for both cluster models. The investigations also showed that the transition energies between each of the three states (Suppl. Table 2) did not strongly depend on the functional group position (R3 or R5). For B-$PhCH_3$ the energy of the first excited state was very low, only about 50 meV above the ground state (we get 0.5 eV for A-$PhCH_3$). Nevertheless, many results are still comparable to the ones for A-$PhCH_3$. From the total energies of each state at the different optimised geometries, we estimate the maximal energy ΔE_{max}, which can be gained during a DIET jump cycle (see Suppl. Table 2).

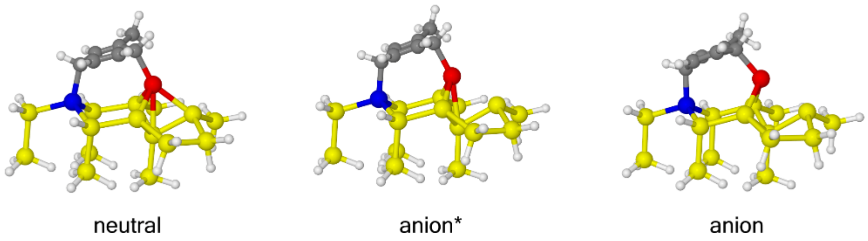

Supplementary Figure 6 Optimised geometries of ground and anion states. Optimised geometries of the neutral, anion* and anion toluene states on the Si(111)-7×7 surface using the A-$PhCH_3$-R3 cluster model. The same colours are used as in Suppl. Fig. 5.

Supplementary Table 1 Calculated bond distances for the optimised geometries. Values are presented in Å. See figure Suppl. Fig. 5 for the atom labels.

		A-PhCH$_3$		B-PhCH$_3$	
		R3	R5	R3	R5
$R_{\mathrm{Si}_a-\mathrm{Si}_d}$	neutral	2.633	2.633	2.639	2.640
	anion*	2.871	2.865	2.678	2.705
	anion	3.029	3.032	3.043	3.043
$R_{\mathrm{Si}_a-\mathrm{Si}_v}$	neutral	2.735	2.734	2.743	2.744
	anion*	2.775	2.773	2.736	2.755
	anion	2.958	2.959	2.973	2.972
$R_{\mathrm{Si}_a-\mathrm{C}_a}$	neutral	2.033	2.033	2.032	2.032
	anion*	2.019	2.021	2.016	2.021
	anion	2.066	2.066	2.064	2.064
$R_{\mathrm{Si}_r-\mathrm{C}_r}$	neutral	2.017	2.017	2.016	2.016
	anion*	2.007	2.007	2.017	2.018
	anion	1.990	1.989	1.988	1.988

Supplementary Table 2 Calculated maximal energy gained during a single DIET jump cycle. Values are presented in eV.

	A-PhCH$_3$		B-PhCH$_3$	
	R3	R5	R3	R5
neutral → anion → neutral	1.146	1.148	1.146	1.143
neutral → anion* → neutral	0.446	0.435	0.174	0.158
neutral → anion* → anion → neutral	1.181	1.176	1.257	1.143

Supplementary Note 6. Density functional theory calculations: transition barriers

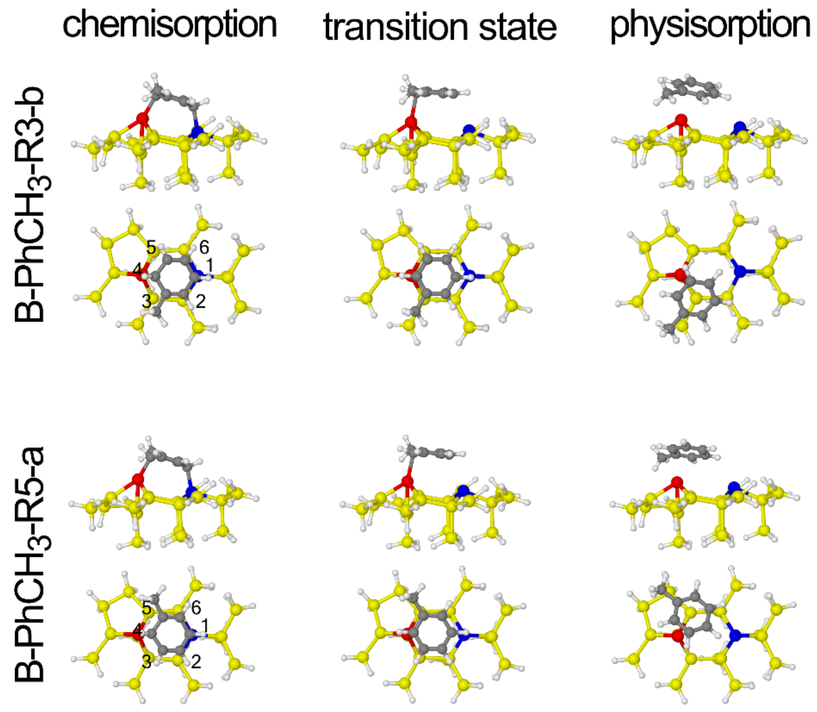

Supplementary Figure 7 Reaction path geometries. Side and top views of geometries for chemisorption (left), the transition states (middle) and physisorption (right) for the reaction path to **B**-$PhCH_3$-R3-b (top rows) and **B**-$PhCH_3$-R5-a (lower rows).

The same methodology for the calculation of barriers and thermodynamic properties was used as in Suppl. Ref. [5] for chlorobenzene. The only difference is that Gaussian16 was used instead of Gaussian09. All calculations were done on the M062X-D3/defTZVP level of theory. Two reaction paths were investigated, i.e, the ones to B-$PhCH_3$-R3-b and B-$PhCH_3$-R5-a, starting from the two most stable positions of the CH3 group Suppl. Ref. [3]. The two other possible paths, i.e., the ones to B-$PhCH_3$-R3-a and B-$PhCH_3$-R5-b (a and b label opposite direction for the displacement of the physisorbed molecule c.f. Suppl. Fig. 7 and Suppl. Ref. [5]), were not considered because here the methyl group would have to pass the adatom. This leads to a large amplitude motion in the lateral direction and possibly to additional barriers, which complicate the identification of a reliable transition state.

The structures of the chemisorbed molecules, the transition states and the physisorbed molecules are shown in Suppl. Fig. 7 for both investigated paths. The main difference to the corresponding paths for B-PhCl is that the transition states are a bit closer to the Si-cluster for the **B**-$PhCH_3$ cases. The distances between the carbon atom and the Si add atom are 2.368 Å and 2.372 Å for B-PhCl-R3-b and B-PhCl-R3-a, while one finds 2.277 Å for both B-$PhCH_3$-R3-b and B-$PhCH_3$-R5-a.

This is also reflected by the energies given in Suppl. Table 3. All barriers are a little bit lower compared to PhCl, e.g., ≈ 0.08 eV for electronic energies. This is mainly due to a lowering of the “earlier” transition states.

Supplementary Table 3 a) Electronic energies, b) Gibbs free energies and c) Kinetic properties of the paths leading to B-PhCH$_3$-R3-b and B-PhCH$_3$-R5-a (C⇒P). Also given in c are the kinetic properties for the transition from physisorption to chemisorption (P⇒C) and from physisorption to the gas phase (P⇒D). All electronic energies and free energies are counterpoise corrected and given in eV relative to the desorbed system.

a	E_{chem}	E_{TS}	E_{physi}	$\Delta E_{\text{chem-TS}}$
B-PhCH$_3$-R3-b	-1.679	-0.342	-0.617	1.337
B-PhCH$_3$-R5-a	-1.676	-0.338	-0.592	1.338

B	G_{chem}	G_{TS}	G_{physi}	$\Delta G_{\text{chem-TS}}$
B-PhCH$_3$-R3-b	-0.883	0.321	-0.049	1.204
B-PhCH$_3$-R5-a	-0.881	0.326	-0.044	1.207

C	A (s^{-1})	E_{A} (eV)	ΔE (eV)
C⇒P			
B-PhCH$_3$-R3-b	$10^{14.91}$	1.329	1.337
B-PhCH$_3$-R5-a	$10^{14.90}$	1.332	1.338
P⇒C			
B-PhCH$_3$-R3-b	$10^{10.93}$	0.260	0.275
B-PhCH$_3$-R5-a	$10^{10.61}$	0.241	0.254
P⇒D			
B-PhCH$_3$-R3-b	$10^{21.96}$	0.591	0.617
B-PhCH$_3$-R5-a	$10^{21.64}$	0.567	0.592

Supplementary References

[1] Y. Cao, J. F. Deng, and G. Q. Xu, *Stereo-Selective Binding of Chlorobenzene on Si(111)-7 × 7*, J. Chem. Phys. **112**, 4759 (2000).

[2] K. R. Rusimova and P. A. Sloan, *Molecular and Atomic Manipulation Mediated by Electronic Excitation of the Underlying Si(111)-7 × 7 Surface*, Nanotechnology **28**, 054002 (2017).

[3] M. Utecht, R. E. Palmer, and T. Klamroth, *Quantum Chemical Approach to Atomic Manipulation of Chlorobenzene on the Si(111)- 7 × 7 Surface: Resonance Localization, Vibrational Activation, and Surface Dynamics*, Phys. Rev. Mater. **1**, 026001 (2017).

[4] M. Utecht, T. Gaebel, and T. Klamroth, *Desorption Induced by Low Energy Charge Carriers on Si(111)-7 × 7: First Principles Molecular Dynamics for Benzene Derivates*, J. Comput. Chem. **39**, 2517 (2018).

[5] T. Gaebel, D. Bein, D. Mathauer, M. Utecht, R. Palmer, and T. Klamroth, *Nonlocal STM Manipulation of Chlorobenzene on Si(111)-7 × 7: Potentials, Kinetics, and First-Principles Molecular Dynamics Calculations for Open Systems*, J. Phys. Chem. C **125**, 12175 (2021).
